# Supervised consumption sites and population-level overdose mortality: a systematic review of recent evidence, 2016–2024

**DOI:** 10.24095/hpcdp.45.9.02

**Published:** 2025-09

**Authors:** Genevive Garipy, Rebecca K. M. Prowse, Rebecca Plouffe, Eva Graham

**Affiliations:** 1 Public Health Agency of Canada, Ottawa, Ontario, Canada; 2 School of Public Health, Universit de Montral, Montral, Quebec, Canada; 3 Dalla Lana School of Public Health, University of Toronto, Toronto, Ontario, Canada

**Keywords:** supervised consumption site, harm reduction, overdose mortality, overdose epidemic, opioids, people who use drugs, PWUD

## Abstract

**Introduction:**

The overdose crisis is one of the most serious public health challenges in North America. Supervised consumption sites (SCSs) effectively prevent onsite overdose deaths and connect people to health services, but their association with population-level overdose mortality remains unclear.

**Methods:**

We searched Embase, Global Health and MEDLINE databases for studies examining associations between SCSs and population-level overdose mortality during the post-2016 overdose crisis (January 2016 to November 2024). Two reviewers, working independently, screened studies, extracted data and assessed study quality using standardized tools (PROSPERO CRD42023406080).

**Results:**

Six studies, all from Canada, met the inclusion criteria. In the four quasi-experimental studies, two large-scale analyses of local health areas or public health units found no significant associations between SCS measures and overdose mortality within provinces. Some analyses of smaller urban areas showed protective associations, although this finding was not consistent across studies. Two observational studies suggested associations between SCS and lower mortality rates, though with methodological limitations.

**Conclusion:**

Province-wide analyses generally did not detect significant associations between areas with and without SCSs and population-level overdose mortality. Analyses suggest that SCSs in some smaller urban contexts were associated with less overdose mortality, though findings were inconsistent. Further research is needed to understand how geographic scale, implementation context and limited service coverage may influence the detection and magnitude of potential effects of SCSs on overdose mortality.

**Highlights**

- In this systematic review we examined evidence from six studies and found mixed associations between supervised consumption sites and population-level overdose mortality.
- Large-scale provincial-level analyses generally found no significant associations between supervised consumption sites and overdose deaths.
- Some studies of smaller geographic areas reported that supervised consumption sites were associated with fewer overdose deaths in certain urban areas, though this finding was not consistent.
- Study design, geographic scale and local implementation context may influence the observed outcomes.

## Introduction

The overdose crisis is one of the most serious public health crises globally and in North America’s recent history. Its escalation in 2016 prompted public health emergency declarations in British Columbia, Virginia, and other regions in North America.^1^,^2^ Between January 2016 and March 2024, Canada recorded 47 162 apparent opioid toxicity deaths, with an annual rate of 21.5 per 100 000 population in 2023.^3^ In the United States, 107 941 opioid overdose deaths were reported in 2022 alone, with an annual rate of 32.4 per 100 000 population.^4^ The COVID-19 pandemic appears to have exacerbated the crisis, as daily apparent opioid toxicity deaths in Canada doubled from 10 in 2019 to 20 in 2022.^3^

Supervised consumption sites (SCSs) represent one of the key public health responses to this crisis.^5^,^6^ SCSs provide safe, accessible and clean spaces for drug consumption. These facilities are staffed with trained personnel who provide harm reduction services and resources, such as safe injecting practices and drug-checking services, and who can intervene during overdose events.^7^ They also connect individuals to health and social services such as substance use treatment and housing supports.^7^ Sites can differ in the consumption modes they supervise (e.g. injection, inhalation, intranasal, oral) and some specialize in particular forms, such as supervised injection facilities. As of 2022, 16 countries had operational SCSs.^5^

Research examining individual-level outcomes indicate multiple benefits among people who use SCS services. Between 2017 and 2024, federally exempted SCSs in Canada responded to more than 60000 overdose events, with no reported onsite fatalities.^8^ Research has also documented social benefits, including improved access to housing and legal and health care services and enhanced community belonging and safety among people who use drugs (PWUD).^8^-^13^ Studies have also observed lower rates of emergency service utilization, fewer nonfatal overdose events, lower all-cause mortality and decreased injection-related complications such as infections and abscesses.^12^-^16^

Despite the documented individual-level benefits of SCSs, the relationship with population-level overdose mortality is less clear. Evaluations from the 2000s show mixed results. After opening in 2003, Vancouver’s Insite, North America’s first sanctioned SCS, was associated with significant reductions in local overdose mortality.^17^ Analysis of Sydney’s Medically Supervised Injecting Centre, Australia’s first such site, found no change in local overdose mortality after its opening in 2001.^18^ Note that both these studies were conducted in a markedly different public health context, before the dramatic rise in overdose deaths that began in 2016.

Subsequent literature reviews have not specifically focused on population-level overdose mortality, and most syntheses drew primarily on the two early studies from Vancouver and Sydney.^12^-^14^,^19^-^22^ The most recent systematic review, covering literature up to 2019, examined injection drug use exclusively.^13^ Since then, the overdose crisis has evolved considerably, shaped by the COVID-19 pandemic, increased amounts of fentanyl and its analogues in the drug supply, and other factors.^23^

Given these evolving conditions and new research examining potential SCS associations with mortality outcomes, an updated systematic review was needed. This study aims to synthesize empirical evidence from 2016 to 2024 to help inform public health responses to the ongoing overdose crisis in the current context.

## Methods

***Systematic review registration***

Our review adhered to the Preferred Reporting Items for Systematic Reviews and Meta-Analyses (PRISMA) 2020 guidelines^24^ and was registered in PROSPERO (CRD42023406080).

***Information sources and search strategy***

We developed a comprehensive search strategy to identify articles in Embase, Global Health and MEDLINE databases published between January 2016 and November 2024. The search terms focused on two main concepts: overdose mortality and SCSs. We restricted our search to English and French publications from 2016 onward to capture literature published during the surge in opioid-related overdose deaths in North America and the changing characteristics of the drug supply.^25^,^26^ Searches were conducted on 20 November 2024. The full search strategies were developed with a librarian. These search strategies are detailed in the supplementary materials (Additional File 1; available from the authors upon request).

***Eligibility criteria***

We included empirical quantitative studies (i.e. observational, quasi-experimental or experimental study designs), published between 1 January 2016 and 11 November 2024, that reported on the association of SCSs with overdose mortality at the population level. Specifically, we included studies that investigated the presence or availability of SCSs, defined as designated spaces that provide onsite monitoring of substance use and rapid response to an overdose event. We included temporary sites, such as overdose prevention sites and urgent public health need sites, which have the same harm reduction function as SCSs but are established on a temporary basis in response to urgent needs in a particular region or community. We also included sites that are limited to a single mode of consumption. We excluded descriptive studies, mathematical modelling studies and those reporting on SCS implementation alone.

We included studies that examined either opioid-related deaths or unspecified overdose deaths, as data from 2023 indicate that most overdose deaths involving other substances also involved opioids.^27^ For example, 81% of accidental apparent stimulant toxicity deaths in Canada also involved opioids.^27^ Because SCSs do not necessarily document the substances used, focusing solely on opioids would have also limited the evidence from SCSs. In Canada, 69% of drugs consumed at SCSs between March 2020 and August 2024 were opioids.^8^

Finally, studies that focused on specific subpopulations (e.g. people experiencing homelessness) were excluded, as our aim was to explore the potential impact of SCSs on the broader population of PWUD. Qualitative research, reviews, editorials, opinion pieces, protocols, case reports, case studies, commentaries and books were also excluded.

***Study selection and data extraction***

After importing references into Covidence (Veritas Health Innovation, Melbourne, AU) and removing duplicates, two reviewers (GG, RKP or RP) independently screened articles against eligibility criteria, first by examining the titles and abstracts and then conducting full-text searches. Discrepancies were resolved through discussion. The same pair of reviewers independently extracted data from included studies, that is, study design, setting, study period, mortality outcome measure, SCS measure, geographical unit of analysis and measures of association (e.g. deaths averted, correlation, regression coefficient). Data extraction discrepancies were resolved through discussion.

***Quality assessment***

We assessed study quality using the JBI critical appraisal tools (JBI, Adelaide, AU)^28^ according to study designs. JBI tools assess risk of bias for observational, quasi-experimental and experimental studies.^28^ Two reviewers (GG, RKP or RP) worked independently to assess the risk of bias, with discrepancies resolved through discussion. Quality assessment forms are provided in Additional File 2 (available from the authors upon request).

***Synthesis methods***

We sorted descriptive and study results into summary tables and summarized findings in a narrative synthesis by study design. We further considered studies with data collected during the COVID-19 pandemic to explore its potential effects on overdose mortality outcomes. Because study design, exposure and outcome measures varied significantly, we did not conduct meta-analyses or meta-regressions.

## Results

***Study selection and characteristics***

**Figure 1:**
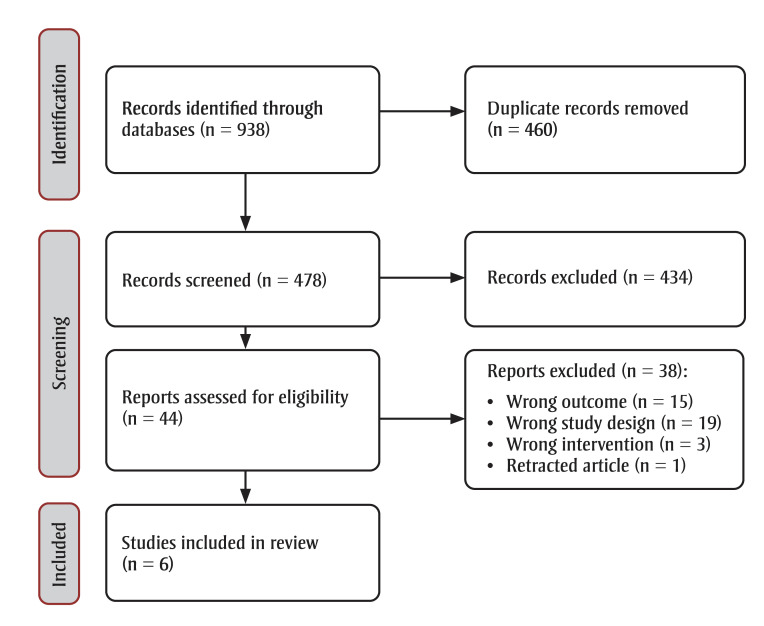
PRISMA 2020 flow chart of the review process

**Abbreviation:** PRISMA, Preferred Reporting Items for Systematic Reviews and Meta-Analyses. 

We included six empirical studies from 478 identified unique references. Of the 44studies retrieved for full-text review, 38 were excluded: 15 did not include the outcome of interest; 19 had the wrong study designs; three did not include the intervention of interest; and one had been retracted (Figure 1).

***Overall characteristics***

Of the six included studies, four were quasi-experimental^29^-^32^ and two were observational.^33^,^34^ All the studies were conducted in Canada. Four focused specifically on opioid overdose deaths,^29^-^32^ one on fentanyl-related overdose deaths^33^ and one on overdose deaths from any substance.^34^ SCSs were operationalized as the implementation of SCSs in four studies,^29^,^31^,^32^,^34^ total visits across SCS locations in one study^33^ and booth-hours per 100000 population in another study.^30^ Three studies included data collected during the COVID-19 pandemic (post March 2020).^30^,^32^,^33^

***Evidence from quasi-experimental studies***

The four quasi-experimental studies used interrupted-time series analysis.^29^-^32^ Two used controlled designs with matched comparisons^29^ or synthetic controls^30^ to distinguish SCS effects from broader changes in overdose mortality, and two examined changes post SCS implementation with no control groups.^31^,^32^ With opioid-related deaths rising across Canada during study periods, uncontrolled analyses would likely underestimate any protective associations with SCSs, as they did not account for increasing mortality trends. The studies revealed varying patterns across jurisdictions, with controlled analyses at provincial levels generally finding no significant associations, while region-specific analyses showed lower overdose mortality rates in certain urban areas (Table 1).

**Table 1:** Characteristics of studies included in the systematic review (n = 6)

Authors, year	Setting	Time period	Mortality outcome	SCS measure	Control group	Geographical unit of analysis	Quantitative measure of association with mortality
Quasi-experimental studies							
Panagiotoglou, 2022^29^	BC, Canada	3 years; 2015–2017	Opioid-related deaths	Local health areas with at least 1 SCS/OPS	Local health areas without an SCS	Local health area	Change in trends of deaths/100,000/month: −0.08; 95% CI: −0.23 to 0.09; *p* = 0.36
Panagiotoglou and Lim, 2022^30^	ON, Canada	7 years; 2014–2021	Opioid-related deaths	SCS/OPS booth-hours per 100,000 population	Synthetic controls that did not have an SCS	PHU	β = 0.000; 95% CI: 0.000 to 0.000; *p* = 0.25
Yeung et al., 2023^31^	Calgary, Edmonton, Red Deer, Lethbridge, AB, Canada	5.5 years; 2013–2019	Opioid-related deaths	Implementation of SCSs/OPSs	None	SCS-service defined local area	Calgary: −1.7 deaths/month; 95% CI: −4.5 to 0.9; *p* = 0.09 Edmonton: −5.9 deaths/month; 95% CI: −8.9 to −2.9; *p* < 0.001 Lethbridge: 0.0 deaths/month; 95% CI: −0.4 to 0.7; *p* = 0.60 Red Deer: −0.1 deaths/month; 95% CI: −0.5 to 0.3; *p* = 0.09
Robinson et al., 2024^32^	ON, Canada	8 years; 2014–2021	Opioid-related deaths	Implementation of SCSs	None	PHU	PHUs with SCSs: +0.02 deaths/100,000/month; *p* = 0.27 PHUs without SCSs: +0.38 deaths/100,000/month; *p* < 0.001
Observational studies							
Marshall et al., 2021^33^	AB, Canada	4 years; 2017–2020	Fentanyl-related deaths	Total number of visits at all 7 provincial SCS/OPS locations	None	Province	*r* = −0.64; *p* = 0.03
Rammohan et al., 2024^34^	Toronto, ON, Canada	2 years; 2017 (1 May–31 July) vs. 2019 (1 May–31 July)	Overdose deaths	Implementation of SCSs/OPSs	None	Neighbourhoods within and beyond 500 m of an SCS	Neighbourhoods within 500 m of an SCS: 67% fewer deaths/100,000; *p* = 0.04 Neighbourhoods beyond 500 m of an SCS: 24% fewer deaths/100,000; *p* = 0.38

**Abbreviations:** AB, Alberta; BC, British Columbia; CI, confidence interval; ON, Ontario; OPS, overdose prevention site; PHU, public health unit; SCS, supervised consumption site. 

Two studies conducted in Ontario used different approaches to analyze data from public health units (PHUs) between 2014 and 2021.^30^,^32^ An analysis that used synthetic controls found no significant association between SCS booth-hours and opioid-related mortality (β = 0.000; 95% confidence interval [CI]: 0.000 to 0.000), though protective effects were observed locally in the PHUs in London (β=−0.004; 95% CI: −0.006 to −0.002) and Thunder Bay (β = −0.004; 95% CI: −0.007 to −0.0002).^30^ A separate study that used an uncontrolled approach found that the PHUs that implemented at least one SCS maintained stable opioid-related mortality rates (+0.02deaths/100 000/month; *p*=0.27), while PHUs without SCSs showed increasing rates (+0.38 deaths/100 000/month;* p*< 0.001), although this difference in trajectories was not directly tested statistically.^32^

An uncontrolled analysis in Alberta examined changes in opioid-related deaths between 2013 and 2019 across four municipalities after the implementation of SCSs.^31^ Edmonton saw the largest change with six fewer deaths per month (−5.9; 95% CI: −8.9 to −2.9), followed by Calgary with two fewer deaths per month (−1.7; 95% CI: −4.5 to 0.9), though the confidence interval indicated uncertainty.^31^ Results from Red Deer (−0.1 deaths/month; 95% CI: −0.5 to 0.3) and Lethbridge (0.0 deaths/month; 95% CI: −0.4 to 0.7) showed no changes.^31^ These declining or stable rates in regions with an SCS occurred during a period when opioid-related deaths across Alberta were increasing.^31^

In British Columbia, a controlled analysis of local health areas that opened SCSs between 2015 and 2017 found no differences in monthly opioid-related mortality rates compared to propensity score-matched controls at the provincial aggregate level (β = −0.08; 95% CI: −0.23 to 0.09).^29^ The study excluded the Downtown Eastside of Vancouver, where Insite is located and where overdose deaths were highest, because an appropriate matched control could not be identified.^29^

Quality assessment indicated low risk of bias for the two studies with control groups^29^,^30^ and higher risk of bias for the two studies without.^31^,^32^ (Additional File 2; available from the authors upon request.)

***Evidence from observational studies***

The two observational studies used ecological study designs to examine associations between SCSs and overdose mortality, one at the province level^33^ and the other at the neighbourhood level^34^ (Table 1). In Alberta, a province-wide analysis found that higher SCS visits across the seven provincial SCSs correlated with fewer fentanyl-related overdose deaths between 2017 and 2020 (r = −0.64; *p* = 0.03).^33^ A study in Toronto, Ontario, compared overdose mortality rates in 2017 and 2019, that is, before and after SCSs were implemented, at different distances from the sites.^34^ Neighbourhoods within 500 m of an SCS had 67% fewer overdose deaths per 100000 people (*p* = 0.04) after the SCSs had been implemented. Areas beyond 500 m of an SCS had 24% fewer deaths, but this difference was not statistically significant (*p* = 0.38).^34^ Quality assessment found that both ecological studies had high risk of bias, primarily because of a lack of control for confounding factors (Additional File 3; available from the authors upon request).

***Evidence from the COVID-19 pandemic***

Three studies included data from before the pandemic, but provided limited insight into pandemic-specific effects.^30^,^32^,^33^ A quasi-experimental analysis of Ontario SCS booth-hours conducted sensitivity analyses excluding pandemic data and found similar nonsignificant impacts on opioid-related mortality.^30^ An ecological study conducted in Alberta reported a 64% decrease in the number of SCS visits and a 118% increase in fentanyl-related overdose deaths during the early months of the pandemic, but did not statistically analyze these patterns.^33^ The other quasi-experimental study from Ontario acknowledged that pandemic-related service changes occurred, but did not assess their impact.^32^ Overall, the influence of the pandemic on SCS operations and population-level overdose mortality remains largely unexplored. 

## Discussion

This systematic review synthesized evidence from six empirical studies examining associations between SCSs and population-level overdose mortality between 2016 and 2024. All studies were from Canada. Of the four quasi-experimental studies, two province-wide analyses of SCSs in local health areas or PHUs found no significant associations. Region-specific analyses yielded mixed results, with lower mortality rates associated with SCSs in some local areas, but not others. Two additional observational studies reported protective associations but had methodological limitations. These studies reveal important nuances in understanding the associations between SCSs and overdose mortality across different contexts, with methodological factors influencing their interpretation.

Geographical scale emerged as a key methodological consideration. The studies that examined smaller geographic units (e.g. neighbourhoods^31^,^34^) were more likely to detect mortality-related associations than the analyses of larger administrative regions. This pattern may reflect both the localized nature of SCS services and implementation factors. Two Ontario studies,^30^,^32^ for example, examined SCSs within PHUs from 630 km to 266 291 km in size.^35^ Examining such a large area could potentially mask localized SCS effects. This aligns with the reports from Toronto^34^ and Vancouver^17^ that SCSs were associated with lower overdose mortality rates within 500 m of the sites but not beyond.

Study design and appropriate controls played a crucial role for interpreting findings. Controlled quasi-experimental analyses provided the strongest evidence by accounting for broader temporal trends in overdose mortality. In this review, the two controlled analyses did not find significant associations at the provincial level between SCSs in local health areas or PHUs and overdose mortality. The interpretation of uncontrolled analyses requires careful consideration of context. During a period when overdose deaths were rising across Canada, stable and even increasing rates in areas with SCSs might suggest potential benefits, as rates could have potentially risen even more rapidly without these services. However, controlled analyses comparing appropriate counterfactuals are needed to test this hypothesis.

Implementation contexts might have also influenced outcomes. The examined sites included established urban SCS programs with strong community support and newer sites in areas with different patterns of substance use and levels of auxiliary services. Facility location and accessibility seem to be key factors. For instance, Edmonton’s centrally located SCS, which is near public transit, had significant reductions in numbers of deaths, while the less central site in Calgary had weaker associations with less precise estimates.^31^ These location-based differences align with qualitative findings from feasibility studies where stakeholders consistently recommend locating SCSs in areas with high levels of drug use, easy access to public transportation and proximity to health facilities.^36^

The potential population-level impacts on mortality may also be limited by the small proportion of total drug consumption that occurs within SCSs. Recent data from Ontario suggest that SCS interventions cover less than 1% of at-risk consumption episodes in the province.^32^ In Vancouver’s Downtown Eastside, where SCS integration is the most extensive in Canada, only 5% of community drug injections occurred under SCS supervision in the early 2000s.^37^ This limited reach is significant given that most overdose fatalities occur in residential settings during solitary use, where SCS services cannot intervene.^38^,^39^

Operational constraints may further restrict potential population-level impacts. These include limited hours of operation, facility capacity restrictions and a lack of specialized services such as supervised inhalation.^38^,^40^,^41^ The scarcity of supervised inhalation services presents a particular challenge, as smoking has become the predominant consumption mode in Canada and, increasingly, the primary method involved in overdose deaths.^42^,^43^ Access barriers such as geographic distance, transportation challenges and stigma may further reduce utilization among PWUD.^11^,^44^-^48^

These findings must be considered within the larger and evolving public health context. The increasing prevalence of fentanyl and its analogues in opioid toxicity deaths,^27^ alongside the growing use of benzodiazepines^49^ and xylazine,^50^ has changed both the risk environment for PWUD and the operational demands on SCS facilities. Available interventions have concurrently expanded to include emerging approaches such as overdose response hotlines and applications, potentially offering broader reach and accessibility to complement facility-based services.^51^,^52^ The COVID-19 pandemic added further complexity through its impact on SCS operations.^53^ The potential association of the pandemic with population-level overdose mortality remains largely unexplored in the current evidence base.

***Limitations of the included studies***

The reviewed studies had some limitations. Most were unable to fully account for concurrent public health interventions, such as naloxone distribution programs, changes in drug supply or changes in service access.^29^,^33^,^54^-^56^ The lack of control groups and group comparisons in some analyses limited the ability to separate SCS-associated changes from underlying overdose mortality trends. While total study periods ranged from 2 to 8 years, the post-SCS implementation periods were much shorter, limiting both the statistical power and ability to evaluate operational programs beyond their initial implementation phases. Studies conducted during the COVID-19 pandemic were challenged by service disruptions.

***Limitations of this review***

Despite SCSs operating in at least 16 countries,^5^ all included studies were from Canada, limiting generalizability. The Canadian context has specific features that may not apply to other jurisdictions, including the federal exemption process for SCSs, universal health care coverage and harm reduction policies.^38^,^53^,^57^ In addition, Canada’s overdose death rates are among the highest globally, comparable only to the United States, reflecting a particularly severe crisis that may not mirror conditions elsewhere.^4^,^5^,^27^

Most of the studied SCSs were in urban settings with high concentrations of overdose deaths, and their associations with mortality outcomes may differ in lower-density areas or regions with fewer overdose deaths.^51^,^58^

This review included only peer-reviewed literature, potentially missing SCS program evaluations and government reports from the grey literature. By focusing on population-level overdose mortality, the review does not address other important benefits of SCSs that can inform policy decisions.

***Future directions***

Several key research priorities should be considered. First, methodological improvements are needed to address current evidence gaps. Future studies should prioritize quasi-experimental designs with appropriate controls to better distinguish SCS-associated changes in population-level mortality from concurrent interventions, changes in drug markets and changes in mortality trends. Research at smaller geographic units of analysis, while accounting for potential spillover effects between regions, could provide clearer insights for local outcomes.

Research on optimizing service delivery represents another critical direction. Studies should examine how different SCS models relate to mortality outcomes across urban, suburban and rural contexts. Research examining specific operational characteristics could further inform service approaches, including permitted consumption modes, responses to polysubstance use, integration with other services (e.g. shelters) and emerging strategies such as mobile and virtual services that could potentially extend service reach.^38^,^52^,^59^ Understanding access barriers remains important, as safety concerns, stigma, the presence of police, inconvenient access and other factors can deter service utilization and impact population-level outcomes.^11^,^44^-^48^

Broader evaluative research could help to guide evidence-based policy decisions. Comprehensive cost-effectiveness analyses that consider both direct and indirect benefits can help capture the full scope of outcomes associated with SCSs.^54^-^56^,^60^ Simulation models incorporating diverse real-world conditions and policy parameters can help explore how site placement, service capacity or complementary interventions might impact population-level mortality.^54^-^56^,^60^-^62^ Research beyond Canada is also essential for understanding how different health care systems and policy contexts relate to overdose mortality outcomes.

## Conclusion

This systematic review revealed mixed evidence for associations between SCSs and population-level overdose deaths. At the provincial level, rigorous quasi-experimental studies found no differences in overdose mortality between local health areas or PHUs with and without SCSs. However, when analyzing specific urban areas and smaller geographic scales, some studies—including those using high-quality methods—found lower mortality rates in regions or neighbourhoods after SCSs were implemented. Although SCSs have well-documented individual-level benefits, their impact on overall population-level mortality is context dependent and less clear. 

SCSs represent one component within comprehensive public health approaches to substance-related harm reduction.57 Their effectiveness may be enhanced by integrating them with other evidence-based interventions, such as the availability of take-home naloxone kits, opioid agonist therapies and drug-checking services.63 This review highlights the need for continued, rigorous research to understand the potential role of SCSs in addressing the overdose crisis.

## Acknowledgements

We would like to thank Shannon Hayes, from Health Canada and the Public Health Agency of Canada health libraries, who helped develop the search term strategies. We also acknowledge our colleagues at the Controlled Substances and Overdose Response Directorate at Health Canada for their review and guidance.

## Funding

This research did not receive any funding from agencies in the public, commercial or not-for-profit sectors.

## Conflicts of interest

None.

## Authors’ contributions and statement

GG: Conceptualization, data curation, formal analysis, investigation, methodology, project administration, supervision, writing—original draft, writing—review and editing.

RKP: Data curation, formal analysis, investigation, methodology, writing—original draft, writing—review and editing.

RP: Conceptualization, formal analysis, investigation, project administration, supervision, writing—original draft, writing—review and editing.

EG: Conceptualization, investigation, project administration, writing—review and editing.

All authors reviewed and approved the final draft of this manuscript.

The content and views expressed in this article are those of the authors and do not necessarily reflect those of the Government of Canada.
