# Supervised consumption sites and population-level overdose mortality: a systematic review of recent evidence, 2016–2024

**DOI:** 10.24095/hpcdp.46.2.04

**Published:** 2026-02

**Authors:** Genevive Garipy, Rebecca K. M. Prowse, Rebecca Plouffe, Eva Graham

**Affiliations:** 1 Public Health Agency of Canada, Ottawa, Ontario, Canada; 2 School of Public Health, Universit de Montral, Montral, Quebec, Canada; 3 Dalla Lana School of Public Health, University of Toronto, Toronto, Ontario, Canada

This corrigendum is being published to add a clarification on page 361 of the following article:

Garipy G, Prowse RKM, Plouffe R, Graham E. Supervised consumption sites and population-level overdose mortality: a systematic review of recent evidence, 2016–2024. Health Promot Chronic Dis Prev Can. 2025;45(9):357-66. https://doi.org/10.24095/hpcdp.45.9.02

Text has been added to highlight an important precision about the Rammohan et al. study^1^. Bold has been used to identify the added text.

The authors thank Dr. Daniel Werb for raising this concern.

## Before correction

The two observational studies used ecological study designs to examine associations between SCSs and overdose mortality, one at the province level^33^ and the other at the neighbourhood level.^34^ In Alberta, a province-wide analysis found that higher SCS visits across the seven provincial SCSs correlated with fewer fentanyl-related overdose deaths between 2017 and 2020 (r = −0.64; p = 0.03).^33^ A study in Toronto, Ontario, compared overdose mortality rates in 2017 and 2019, that is, before and after SCSs were implemented, at different distances from the sites.^34^ Neighbourhoods within 500 m of an SCS had 67% fewer overdose deaths per 100 000 people (p=0.04) after the SCSs had been implemented. Areas beyond 500 m of an SCS had 24% fewer deaths, but this difference was not statistically significant (p = 0.38).^34^ Quality assessment found that both ecological studies had high risk of bias, primarily because of a lack of control for confounding factors (Additional File 3; available from the authors upon request).

## After correction

The two observational studies used ecological study designs to examine associations between SCSs and overdose mortality, one at the province level^33^ and the other at the neighbourhood level.^34^ In Alberta, a province-wide analysis found that higher SCS visits across the seven provincial SCSs correlated with fewer fentanyl-related overdose deaths between 2017 and 2020 (r = −0.64; p = 0.03).^33^ A study in Toronto, Ontario, compared overdose mortality rates in 2017 and 2019, that is, before and after SCSs were implemented, at different distances from the sites.^34^ Neighbourhoods within 500 m of an SCS had 67% fewer overdose deaths per 100 000 people (p=0.04) after the SCSs had been implemented. Areas beyond 500 m of an SCS had 24% fewer deaths, but this difference was not statistically significant (p = 0.38).^34^** The study also conducted geographically weighted regression analyses of post-implementation spatial patterns, finding inverse associations between SCS proximity and overdose mortality that strengthened from 2018 to 2019. These analyses adjusted for neighbourhood-level sociodemographic factors and substance-use-related health services.** Quality assessment found that both ecological studies had high risk of bias, primarily because of a lack of control for confounding factors (Additional File 3; available from the authors upon request).

## References

[B01] Rammohan I, Gaines T, Scheim A, Bayoumi A, Werb D (2024). Overdose mortality incidence and supervised consumption services in Toronto, Canada: an ecological study and spatial analysis. Lancet Public Health.

